# Dietary Soy Isoflavone Alleviates Oxidized Fish Oil-Induced Growth Inhibition and Hepatic Injury in Rice Field Eel (*Monopterus albus*): Involvement of Antioxidant Capacity, Digestive Function, Endoplasmic Reticulum Stress, and Inflammation

**DOI:** 10.3390/ani15192839

**Published:** 2025-09-29

**Authors:** Quan Li, Huahong Wei, Tao Zhou, Kai Xie, Yi Hu, Junzhi Zhang

**Affiliations:** Fisheries College, Hunan Agricultural University, Changsha 410128, China

**Keywords:** soybean isoflavone, oxidized fish oil, oxidative stress, endoplasmic reticulum stress, inflammation, rice field eel

## Abstract

An 8-week feeding trial was conducted to evaluate the potential of dietary soy isoflavones (SIF) to counteract growth inhibition, oxidative stress, endoplasmic reticulum stress, and inflammation induced by oxidized fish oil in *Monopterus albus* (initial body weight: 26.0 g). The fish were reared in outdoor pond-based net cages (2.0 m × 1.5 m × 1.5 m; 3 cages per treatment, 50 fish per cage) and fed five isonitrogenous and isolipidic experimental diets: a control diet (CON) containing 2.2% fresh fish oil, and oxidized fish oil diets (2.2% oxidized oil) supplemented with 0 (SIF0), 25 (SIF25), 50 (SIF50), or 100 (SIF100) mg/kg SIF. The fish were fed to satiation daily at 4:00 pm at a rate of 3–5% of initial body weight. Compared with the CON group, the SIF0 group showed significantly reduced growth performance, with a final weight gain rate of 84.13%, and decreased intestinal digestive enzyme activity. Hepatic superoxide dismutase (SOD) and catalase (CAT) activities declined to 1.78 U/mgprot and 4.13 U/mgprot, respectively, while serum glutamic-oxaloacetic transaminase (GOT) and glutamic-pyruvic transaminase (GPT) levels increased to 24.95 U/L and 37.56 U/L. The SIF0 diet also up-regulated the expression of hepatic endoplasmic reticulum stress-related genes (*perk*, *ire1*, *atf6*) and pro-inflammatory genes (*tnfα*, *il-1β*). Dietary SIF supplementation alleviated these adverse effects, with the most pronounced improvements observed at 50 mg/kg SIF based on overall performance. These results demonstrate that SIF can serve as a functional feed additive to mitigate oxidative stress and related metabolic disorders in *M. albus*.

## Simple Summary

The inclusion of oxidized fish oil in aquafeeds can have detrimental effects on farmed fish, including growth inhibition and physiological stress. This study aimed to evaluate the potential of dietary soy isoflavones (SIF) to mitigate the adverse effects of oxidized fish oil in *Monopterus albus*. Our findings indicate that prolonged consumption of a diet containing oxidized fish oil significantly reduced intestinal digestive enzyme activity, suppressed growth, and induced hepatic oxidative stress, endoplasmic reticulum stress, inflammation, and subsequent liver damage. However, dietary supplementation with SIF effectively counteracted these negative outcomes. These results suggest that SIF is a promising functional feed additive in aquaculture that can alleviate the harmful effects associated with lipid oxidation in aquafeeds, thereby supporting the health and farming of *Monopterus albus*.

## 1. Introduction

As one of the three fundamental macronutrients, lipids are crucial for aquatic animals, not only for their protein-sparing effect but also for vital physiological functions, including acting as carriers for fat-soluble vitamins and participating in the construction of cell membranes [1]. Currently, fish oil (FO), rich in highly unsaturated fatty acid (HUFA), particularly eicosapentaenoic acid (EPA) and docosahexaenoic acid (DHA), remains an indispensable fat source in aquaculture [2]. Building upon this, extensive research has demonstrated that n-3 HUFA significantly promotes fish growth, maintains bodily antioxidant homeostasis, and enhances immune function [3,4,5]. However, the presence of unsaturated bonds makes these fatty acids susceptible to degradation upon catalysis by external factors such as ultraviolet light and strong light, generating unstable free radicals [6]. These radicals then combine with oxygen, initiating a chain reaction of peroxidation that continues until the unsaturated fatty acids are completely oxidized, leading to the complete rancidity of the fish oil [7]. This phenomenon frequently occurs in practical production due to improper feed storage. The consumption of oxidized fish oil (OFO) has numerous deleterious effects on fish. In grass carp (*Ctenopharyngodon idella)* [8] and yellow catfish (*Pelteobagrus fulvidraco*) [9], OFO has been observed to trigger oxidative stress, impair intestinal structure, and subsequently impede growth.

As a vast and fluid network of membranes in eukaryotic cells, endoplasmic reticulum (ER) is integral to cellular equilibrium. Its responsibilities are multifaceted, encompassing protein maturation processes like synthesis and folding, alongside its critical role in regulating intracellular calcium dynamics [10]. Nevertheless, a state of redox imbalance within an organism can cause an accumulation of reactive oxygen species (ROS) in the ER lumen, intensifying its oxidative milieu. Such alterations impede the correct formation of disulfide bonds, causing a buildup of improperly folded proteins that instigates ER stress (ERS) [11]. Moreover, prolonged ERS can drive liver inflammation and result in hepatic damage via the engagement of inflammatory pathways and the inflammasome [12].

Soy isoflavones (SIF)—primarily genistein, daidzein, and glycitein—are flavonoid secondary metabolites produced by leguminous plants. SIFs possess intrinsic antioxidant capabilities stemming from their chemical structure; specifically, the hydroxyl groups on their phenolic rings can neutralize free radicals by donating hydrogen atoms [13]. In addition to this direct mechanism, SIFs exhibit indirect antioxidant activity. Their structural resemblance to estrogen classifies them as phytoestrogens [14] and enables them to activate estrogen receptors. This receptor activation is known to confer cellular protection against oxidative stress, for instance, by maintaining mitochondrial function in cardiomyocytes to prevent cell death [15]. Evidence from various animal models confirms the antioxidant effects of SIF. In broiler chickens, dietary SIF curbed lipid peroxidation and enhanced overall antioxidant capacity [16]. Analogous effects have been observed in aquaculture, where supplementing the diet of juvenile golden pompano (*Trachinotus ovatus*) with 40 mg/kg of SIF boosted hepatic CAT activity and decreased MDA levels [17].

The rice field eel (*Monopterus albus*, *M. albus*) is a commercially significant carnivorous fish in China’s aquaculture industry, with national production reaching over 350,000 tons in 2023 (National Bureau of Statistics of China, 2024) [18]. However, a significant challenge in its cultivation is the high susceptibility of powdered formulated feeds to oxidation—due to their larger specific surface area compared to pelleted feeds [19]. This accelerated oxidation poses substantial health risks, particularly to the liver, which is the central organ for lipid metabolism and highly vulnerable to damage from dietary oxidized lipids [20]. These concerns highlight the urgent need for effective nutritional interventions to mitigate oxidative damage and associated growth retardation in this species.

## 2. Materials and Methods

### 2.1. Experimental Feeds

Feed ingredients used were fish meal, soy protein concentrate, and poultry by-product meal as protein sources, and fish oil (FO) and oxidized fish oil (OFO) as lipid (oil) sources (Table 1). The test diets were formulated to be isonitrogenous and isolipidic, complying with the previously estimated optimal dietary protein and lipid levels for *M. albus* [21]. To induce accelerated oxidation, fresh FO with a starting peroxide value (POV) of 5 meq O_2_/kg was heated in a 60 °C water bath. The process was facilitated by continuous oxygen bubbling and simultaneous irradiation from an ultraviolet lamp. The progression of oxidation was tracked by periodically measuring the POV, and the procedure was terminated when a final POV of 831 meq O_2_/kg was achieved, indicating the oil was completely oxidized. The POV was measured according to the standard iodometric titration method (AOCS Cd 8b-90) [22]. Based on this, five groups of isonitrogenous and isofat feeds were formulated in this experiment: the group without added SIF and using fresh FO as the control group (CON); the group without added SIF but using OFO as the negative control group (SIF0); and on the basis of the feeds of the SIF0 group, SIF was added at 25, 50, and 100 mg/kg, respectively, which formed the experimental groups SIF25, SIF50 and SIF100. According to the feed formula of each group, the ingredients were weighed accurately and mixed well to make powdered feed. The prepared feeds were stored in a refrigerator at −20 °C.

The proximate composition of the experimental diets was analyzed using standardized methods. Feed samples were first dried in a forced-air oven at 105 °C until constant weight. Crude fat content was determined using the Soxhlet extraction method [23]. A precisely weighed aliquot of the dried sample was wrapped in quantitative filter paper and subjected to reflux extraction with petroleum ether in a Soxhlet apparatus. Following extraction, the residual sample package was dried to a constant weight. The amount of crude fat was quantified by measuring the sample’s mass difference pre- and post-extraction. Concurrently, the Kjeldahl procedure was employed to ascertain the crude protein level [24]. A separate aliquot of the dried sample underwent catalytic digestion with concentrated sulfuric acid for 4 h. The total nitrogen content was subsequently measured using a Kjeldahl analyzer. The percentage of crude protein was obtained from the product of the total nitrogen content (%) and the conventional conversion factor of 6.25.

One hour before feeding, the feed was taken out and thawed, and then kneaded into dough by adding appropriate amount of water.

### 2.2. Feeding Management

The experimental rice field eels were purchased from Xihu Eel Farming Base, Hanshou, Changde, China. After a 2-week acclimatization period, M. albus were selected to fast for 24 h and randomly assigned to 15 floating net cages (2.0 m × 1.5 m × 1.5 m) in outdoor ponds, all of uniform specification. Following the fasting period, all fish in each cage were bulk-weighed, and the total weight was divided by the number of fish to obtain the initial body weight (IBW) per fish. The initial cage biomass was calculated as the total weight of all fish stocked in each cage. A total of five treatment groups (CON, SIF0, SIF25, SIF50, SIF100) were set up in the experiment, and each treatment group was set up with three replicated nets, with 50 eels stocked in each net cage, totaling 750 *M. albus*. Prior to formal feeding of the experimental diets, a one-week period to acclimate the eels to the experimental feeding regime was conducted using a mixture of fresh earthworms and fish slurry. The formal feeding period lasted for 8 weeks, and the feeding rate was 3%~5% of the initial body weight of the eels at 4:00 p.m. every day. The amount of feed consumed was recorded daily by weighing the feed offered and subtracting the uneaten feed collected 2 h after feeding. Satiation was determined when *M. albus* stopped actively feeding. The biomass in each cage was estimated every two weeks by batch-weighing all eels in the cage to adjust the feeding amount. Throughout the entire rearing period, water temperature and other physicochemical parameters were maintained within ranges suitable for the species. The mean water temperature was 29.0 °C, pH was kept between 7.1 and 7.5, ammonium concentration remained below 0.5 mg/L, and dissolved oxygen levels were consistently maintained above 6.5 mg/L.

### 2.3. Sample Collection

At the conclusion of the culture period, the total number and batch weight of *M. albus* in each net cage were recorded. Subsequently, 12 individuals were randomly selected from each net cage, their body length and weight were measured, and blood samples were collected from the caudal vein using a 2 mL syringe. The collected blood samples were placed on ice and allowed to fully stratify, then centrifuged at 3000 rpm for 10 min, and the supernatant was aspirated to obtain serum samples. Concurrently, the same *M. albus* were dissected to excise the entire liver and a section of the foregut tissue. All collected samples (serum, liver, and foregut tissue) were transferred to sterile, enzyme-free centrifuge tubes and stored at −80 °C pending further analysis. The specific growth metrics is as follows: IBW (g): Initial body weight; FBW (g): Final body weight; WGR (%): Weight gain rate = 100 × (FBW − IBW)/IBW; SR (%): Survival rate = 100 × final number/initial number; SGR (%/d): Specific growth rate = 100 × [ln(FBW) − ln(IBW)]/days; CF (100 g/cm^3^): Condition factor = 100 × body weight/(body length)^3^;

### 2.4. Histological Preparation and Staining

Following fixation, liver tissue samples (approximately 1.0 cm × 1.0 cm × 0.3 cm in size) collected from one randomly selected fish per cage underwent three washing cycles with phosphate-buffered saline. Dehydration was then accomplished using a graded ethanol series, after which the tissues were cleared in xylene and embedded in paraffin wax. Finally, the paraffin blocks were cut into 5 μm thick sections and stained with H&E for microscopic qualitative evaluation, including hepatocellular structure, nuclear morphology, and the presence and extent of vacuolation.

### 2.5. Serum Biochemistry Indices Measurement

For the analysis of serum biochemistry, two serum samples were randomly selected from each cage, retrieved from −80 °C storage, and thawed on ice. The concentrations of Triglyceride (TG), Total Cholesterol (TC), Total Bile Acids (TBA), Glutamic-oxaloacetic Transaminase (GOT), Glutamic-pyruvic Transaminase (GPT), High-density Lipoprotein Cholesterol (HDLC), and Low-density Lipoprotein Cholesterol (LDLC) were quantified. All parameters were assayed using commercial kits procured from the Nanjing Jiancheng Bioengineering Institute (Nanjing, Jiangsu, China), in strict accordance with the manufacturer’s protocols.

### 2.6. Biochemical Indices of Tissues

Two liver and two intestine tissue samples per net cage were retrieved from −80 °C storage and thawed on ice. For each sample, a 0.1 g portion was precisely weighed and transferred to a sterile, nuclease-free tube. The tissue was then homogenized in 9 volumes (0.9 mL) of pre-chilled physiological saline to produce a 10% (*w*/*v*) homogenate. The homogenization process was conducted on an ice bath to prevent enzyme degradation. The resulting homogenates were clarified by centrifugation at 3000× *g* for 10 min at °C. The tissue homogenate supernatants were used to quantify the levels of Lipase (LPS), Amylase (AMS), Trypsin (TRY), and a panel of antioxidant-related biomarkers: Catalase (CAT), Superoxide Dismutase (SOD), Glutathione (GSH), Malondialdehyde (MDA), Hydrogen Peroxide (H_2_O_2_), and Total Antioxidant Capacity (T-AOC). All determinations were performed using commercial assay kits in strict accordance with the manufacturers’ protocols. The kit for TRY was procured from Jianglai Biologicals Co. (Shanghai, China), while all other kits were purchased from the Nanjing Jiancheng Bioengineering Institute (Nanjing, China).

### 2.7. Reverse Transcription and Real-Time PCR

Two liver samples (approximately 0.5 cm × 0.5 cm × 0.5 cm) from each net cage were retrieved from a −80 °C freezer. Total RNA was extracted from the liver tissue using the TRIzol reagent method. The extracted RNA was first evaluated for both concentration and integrity. Integrity was confirmed by observing distinct 28S and 18S ribosomal RNA bands with minimal degradation on a 1% agarose gel. The cDNA was synthesized from the qualified RNA samples using the Hifair^®^ III 1st Strand cDNA Synthesis Kit (YEASEN, Shanghai, China). Subsequently, the synthesized cDNA served as the template for qPCR amplification. All qPCR reactions were carried out using the Hieff^®^ qPCR SYBR^®^ Green Master Mix (YEASEN) on a real-time thermal cycler. The qPCR amplification was performed under the following conditions: initial denaturation at 95 °C for 5 min, 40 cycles of denaturation at 95 °C for 10 s, and annealing/extension at 60 °C for 30 s. A melt curve analysis was added to confirm the specificity of the amplification. Gene expression was calculated using the 2^(−ΔΔCt)^ method. The primer sequences for the target genes are provided in Table 2, with rpl17 serving as the internal reference.

### 2.8. Data Processing

The normality and homogeneity of variance for all data were first evaluated using the Kolmogorov–Smirnov and Levene’s tests, respectively. A one-way ANOVA with Tukey’s HSD post hoc test was subsequently used for normally distributed data with equal variances. The Kruskal–Wallis test, followed by Dunn’s post hoc test, was employed for data that did not meet these criteria. For all analyses, significance was accepted at *p* < 0.05, and data are expressed as the mean ± standard error (SEM) with the cage as the experimental unit (*n* = 3). Statistical analyses were carried out using SPSS software (version 26.0), while Python (version 3.10.7) was used for figure generation.

## 3. Results

### 3.1. Growth Result of M. albus

While no statistical difference in SR was found among the groups (*p* = 0.08), the SIF0 group displayed a marked decrease in FBW, WGR, and SGR (*p* < 0.05). The addition of SIF, however, markedly elevated FBW, WGR, SGR, and CF when compared to the SIF0 group (*p* < 0.05). Notably, the SIF50 group demonstrated the highest FBW, WGR, SGR, and CF (Table 3).

### 3.2. Analysis of Serum Biochemical Indices

**Figure 1:**
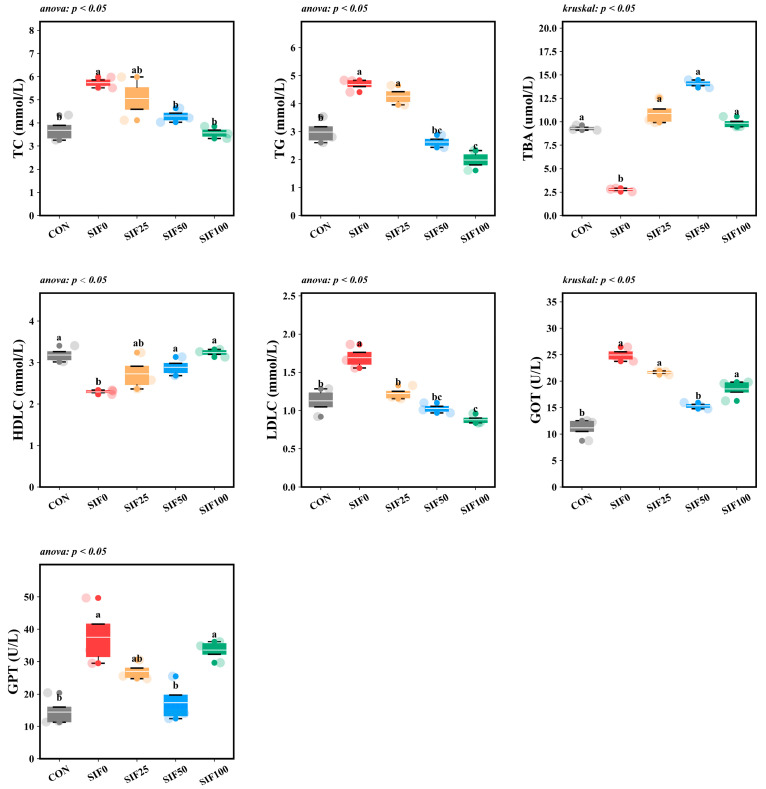
Analysis of serum biochemical indicators (*n* = 3). Note: In the same row, different superscripted lowercase letters indicate significant differences (*p* < 0.05). TC (mmol/L): Total Cholesterol; TG (mmol/L): Triglyceride; TBA (μmol/L): Total Bile Acid; HDLC (mmol/L): High-density Lipoprotein Cholesterol; LDLC (mmol/L): Low-density Lipoprotein Cholesterol; GOT (U/L): Glutamic-oxaloacetic transaminase; GPT (U/L): Glutamic-pyruvic transaminase.

Compared to the CON group, SIF0 group markedly elevated the serum concentrations of TC, TG, LDLC, GOT, and GPT in *M. albus* (*p* < 0.05), while TBA and HDLC levels were considerably reduced (*p* < 0.05). Following the administration of the SIF-supplemented diets, the SIF-supplemented groups exhibited significantly lower serum concentrations of TC, TG, LDLC, GOT, and GPT compared to the SIF0 group (*p* < 0.05), whereas TBA, and HDLC concentrations were significantly increased (*p* < 0.05). Notably, a clear dose-dependent reduction in TC and TG was observed, with the SIF100 group achieving the lowest concentrations of both (Figure 1).

### 3.3. Intestinal Digestive Enzyme Activity

**Figure 2:**
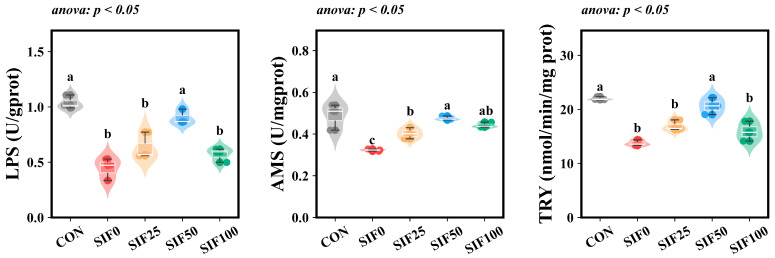
Analysis of intestinal digestive enzymes (*n* = 3). Note: In the same row, different superscripted lowercase letters indicate significant differences (*p* < 0.05). LPS (U/gprot): Lipase; AMS (U/mgprot): Amylase; TRY (nmol/min/mgprot): Trypsin.

Compared to the CON group, the intestinal activities of LPS, AMS, and TRY in *M. albus* from the SIF0 were significantly decreased (*p* < 0.05). However, following SIF supplementation in the feed, the intestinal activities of LPS, AMS, and TRY were notably elevated when compared to the SIF0 (*p* < 0.05). Among the SIF-supplemented groups, the SIF50 group exhibited the highest activities for LPS, AMS, and TRY (Figure 2).

### 3.4. Histomorphological Analysis of the Liver

**Figure 3:**
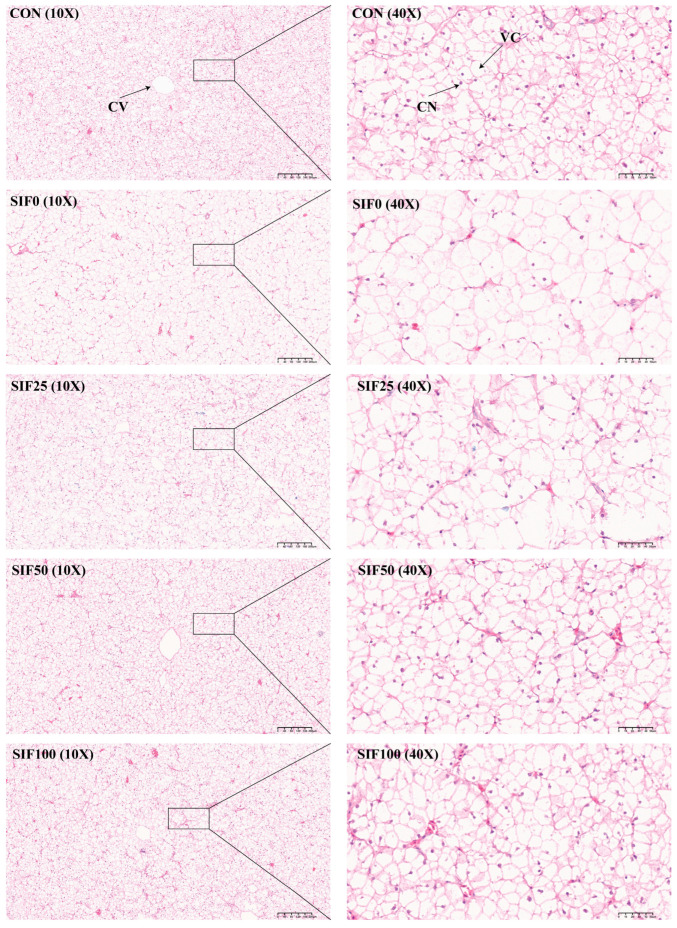
Histological Analysis of Liver Tissue (*n* = 3). Note: CV: Central veins (the veins in the center of the hepatic lobule are surrounded by radial liver cells, which are important vessels for the exchange of substances between the liver and other organs); CN: Cell nucleus; VC: Vacuolization cell.

Figure 3 illustrates representative histological changes in the liver. Compared to the CON group, liver sections from the SIF0 group exhibited morphological alterations, including an apparent reduction in nuclei, enhanced vacuolation, and indistinct hepatocellular boundaries. Dietary SIF supplementation ameliorated these alterations, with SIF-treated groups showing improved nuclear visibility and reduced vacuolation compared to the SIF0 group.

### 3.5. Biochemical Indicators of Hepatic Antioxidation

**Figure 4:**
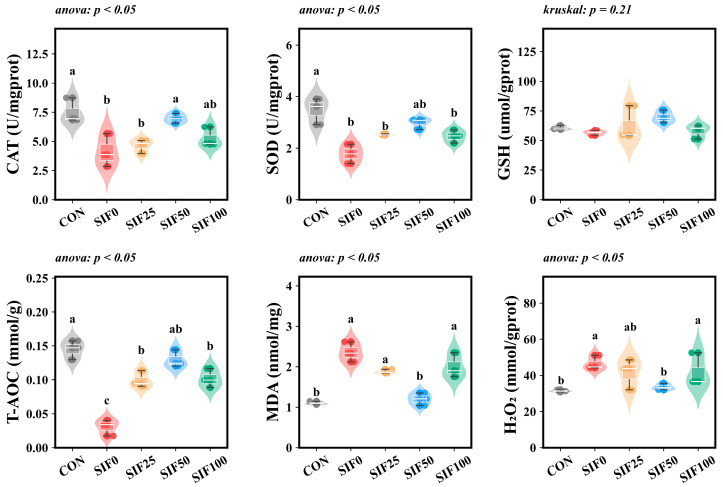
Analysis of hepatic antioxidant biochemical indexes (*n* = 3). Note: In the same row, different superscripted lowercase letters indicate significant differences (*p* < 0.05). CAT (U/mgprot): Catalase; SOD (U/mgprot): Superoxide Dismutase; GSH (μmol/prot): Glutathione; T-AOC (mmol/g): Total Antioxidant Capacity; MDA (nmol/mg): Malondialdehyde; H_2_O_2_ (mmol/gprot): Hydrogen Peroxide.

No statistical difference was found in hepatic GSH concentration among the groups (*p* = 0.08). Compared to the CON group, the SIF0 group exhibited significantly decreased activities of CAT and SOD, as well as a lower T-AOC level (*p* < 0.05). Conversely, the hepatic contents of MDA and H_2_O_2_ were significantly enhanced (*p* < 0.05). Following SIF supplementation, the SIF50 group showed an increase in hepatic SOD activity when compared to the SIF0 group. Furthermore, the SIF50 group demonstrated significantly increased CAT activity and T-AOC levels (*p* < 0.05), coupled with notably lower contents of MDA and H_2_O_2_ (*p* < 0.05) (Figure 4).

### 3.6. Relative Expression of Hepatic Antioxidant Genes

**Figure 5:**
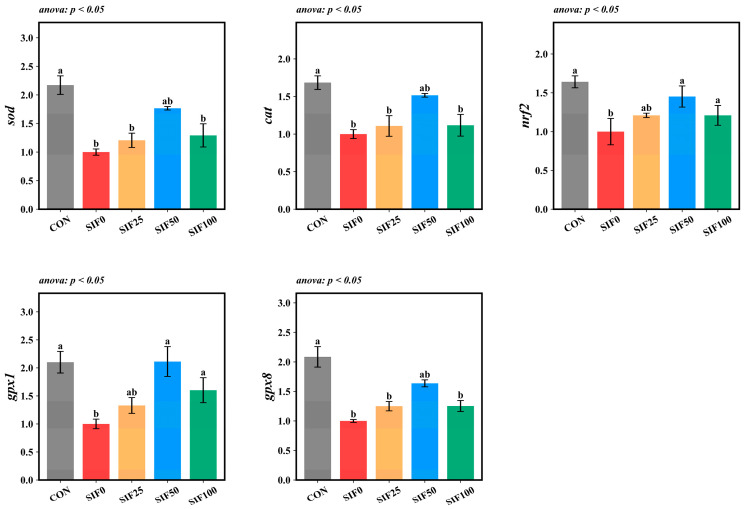
Analysis of hepatic antioxidant gene expression *(n* = 3). Note: In the same row, different superscripted lowercase letters indicate significant differences (*p* < 0.05). *sod*: superoxide dismutase; *cat*: catalase; *nrf2*: nuclear factor erythroid 2-related factor; *gpx1*: glutathione peroxidase 1; *gpx8*: glutathione peroxidase 8.

The OFO diet (SIF0 group) substantially suppressed the hepatic expression of key antioxidant genes—*sod*, *cat*, *nrf2*, *gpx1*, and *gpx8*—when compared to the CON group (*p* < 0.05). Following dietary supplementation with SIF, the expression of *sod*, *cat*, and *gpx8* was upregulated relative to the SIF0 group, while the expression of *nrf2* and *gpx1* was significantly upregulated (*p* < 0.05). Notably, among all SIF-supplemented groups, the highest transcript levels for all five genes were consistently observed in the SIF50 group (Figure 5).

### 3.7. Hepatic Endoplasmic Reticulum Stress Gene Relative Expression

**Figure 6:**
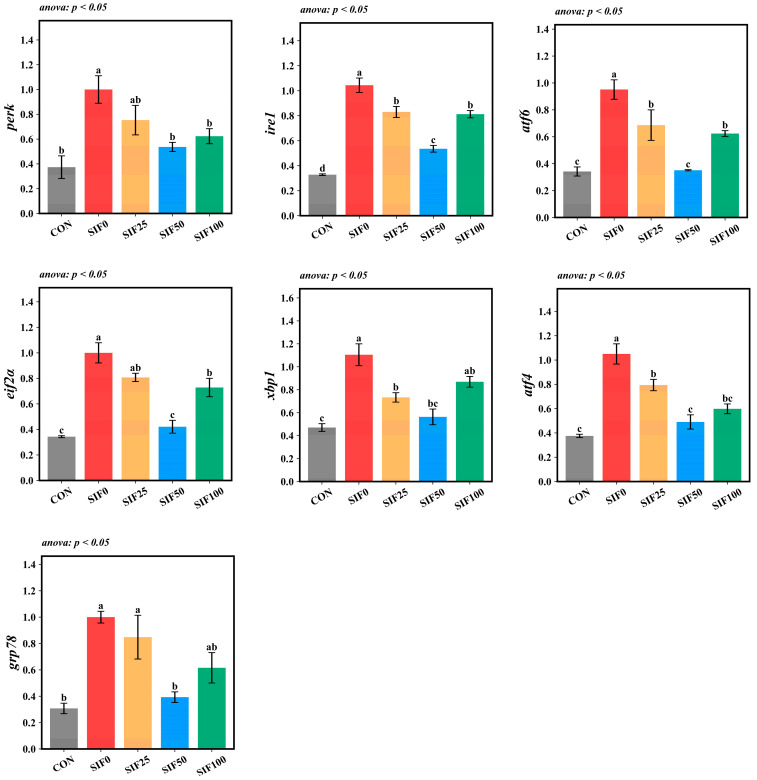
Analysis of endoplasmic reticulum stress gene expression (*n* = 3). Note: In the same row, different superscripted lowercase letters indicate significant differences (*p* < 0.05). *perk*: protein kinase r-like endoplasmic reticulum kinase; *ire1*: inositol-requiring enzyme 1; *atf6*: activating transcription factor 6; *eif2α*: eukaryotic initiation factor-2α; *xbp1*: x-box binding protein 1; *atf4*: activating transcription factor 6; *grp78*: glucose-regulated protein 78.

The hepatic mRNA abundance of ERS-related genes—*perk*, *ire1*, *atf6*, *eIf2α*, *xbp1*, *atf4*, and *grp78*—was significantly elevated in the SIF0 group relative to the CON group (*p* < 0.05). In contrast, SIF supplementation counteracted this upregulation, lowering the expression of all measured genes compared to the SIF0 group (*p* < 0.05). The most pronounced downregulation was consistently seen in the SIF50 group (Figure 6).

### 3.8. Relative Expression of Hepatic Inflammatory Genes

**Figure 7:**
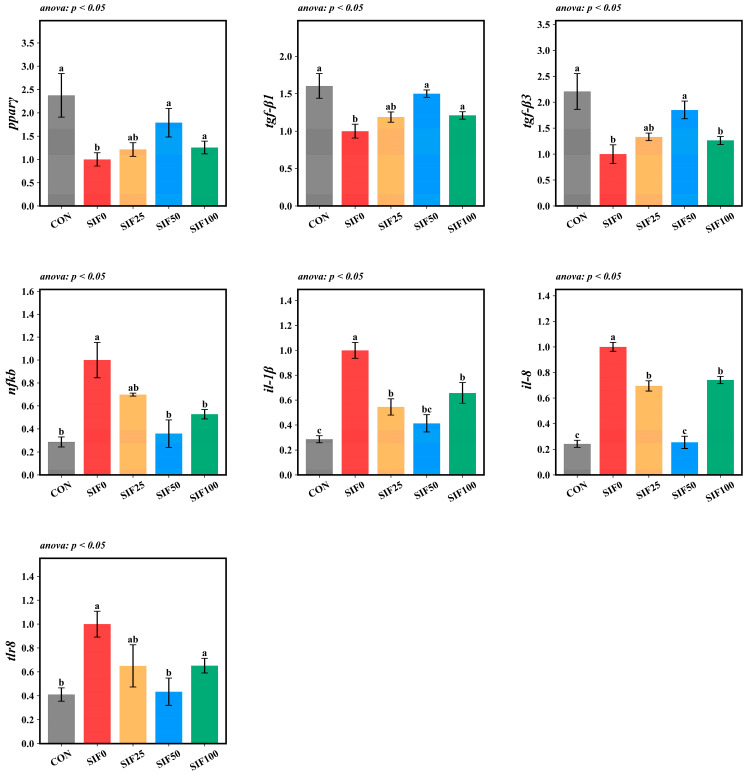
Analysis of hepatic inflammatory gene expression (*n* = 3). Note: In the same row, different superscripted lowercase letters indicate significant differences (*p* < 0.05). *pparγ*: peroxisome proliferator-activated receptor gamma; *tgf-β1*: transforming growth factor beta 1; *tgf-β3*: transforming growth factor beta 3; *nfkb*: nuclear factor kappa b; *il-1β*: interleukin-1 beta; *il-8*: interleukin-8; *tlr-8*: toll-like receptor 8.

Compared to the CON group, the SIF0 group exhibited significantly downregulated relative hepatic expression of *pparγ*, *tgf-β1*, and *tgf-β3* (*p* < 0.05), whereas the relative hepatic expression of *nfkb*, *il-1β*, *il-8*, and *tlr8* was significantly upregulated (*p* < 0.05). In comparison with the SIF0 group, an opposite trend was observed for these genes in the SIF-supplemented groups (*p* < 0.05), with the SIF50 group showing the greatest magnitude of change (Figure 7).

## 4. Discussion

In aquaculture, growth performance serves as the most direct metric for evaluating economic value. Chronic dietary intake of OFO led to a significant reduction in the growth performance of *M. albus* in this study. This finding is consistent with results from studies on the *Pelteobagrus fulvidraco* [9] and the hybrid grouper (*Epinephelus fuscoguttatus ♀ × Epinephelus lanceolatus ♂*) [25], supporting the notion that OFO is detrimental to a range of fish species. However, it is noteworthy that previous research by our team on *M. albus* yielded a different conclusion [26]. This discrepancy may be attributed to several factors, including the inclusion level of the OFO, its degree of oxidation, and the initial size of the experimental fish. The potential mechanisms underlying this growth inhibition may involve two aspects: Firstly, the strong off-flavors generated by volatile compounds such as aldehydes and ketones during oxidation can reduce feed palatability, thereby affecting feed intake [27]. Secondly, toxic metabolites, including small-molecule aldehydes and ketones produced during the oxidation process, can diminish the nutritional value of the diet [28]. Encouragingly, this study revealed that supplementing the OFO with SIF effectively ameliorated the growth performance of *M. albus*. This beneficial effect has also been reported in studies on the Chinese mitten crab (*Eriocheir sinensis*) [29] and the *Trachinotus ovatus* [17]. The functional level of intestinal digestive enzymes is a key determinant of nutrient digestion and absorption efficiency, which in turn dictates an organism’s growth performance [30]. Our experiment found that long-term feeding with OFO markedly reduced activity of intestinal digestive enzymes in *M. albus*, whereas supplementation with SIF mitigated this negative effect. Thus, SIF may promote growth in *M. albus* by ameliorating the adverse impacts of OFO on digestive enzymes.

It is worth noting that the basal diet contained 40% FO, which likely supplied sufficient essential fatty acids to meet the nutritional requirements of *M. albus*. Therefore, the OFO primarily functioned as an oxidative stressor rather than a nutritional source, and the beneficial effects of SIF are likely attributable to its antioxidant properties rather than compensation for nutritional deficiency.

TG and TC levels are not only regulated by exogenous dietary intake but are also critically controlled by endogenous synthesis in the liver, making their fluctuations effective indicators of the body’s lipid metabolic status [31]. In this study, we found that long-term feeding with a diet containing OFO caused a significant buildup of TC and TG in the serum of the *M. albus*. This result is consistent with findings reported by Di Liu in orange-spotted grouper (*Epinephelus coioides*) [32] and by Samad Rahimnejad in rainbow trout (*Oncorhynchus mykiss*) [33]. HDLC and LDLC are the primary carriers for cholesterol transport in the blood [34]. Meanwhile, TBA plays a vital role in fat emulsification, maintaining cholesterol solubility, and lipid transport, thereby participating in fat and cholesterol metabolism [35]. In our experiment, we observed that prolonged dietary exposure to OFO substantially lowered serum HDLC and TBA levels while elevating LDLC content. These changes may collectively impair the body’s capacity for metabolic clearance of TC and TG, leading to their accumulation in the serum. Furthermore, previous studies have shown that OFO can induce oxidative stress and mediate mitochondrial dysfunction, ultimately resulting in the dysregulation of lipid metabolism [36]. Notably, with the dietary supplementation of SIF, serum TC, TG, and LDLC levels decreased, whereas HDLC and TBA levels increased. This suggests that SIF may effectively ameliorate the lipid accumulation induced by OFO, possibly by enhancing the efficiency of reverse cholesterol transport to the liver and accelerating the clearance of TC and TG. Moreover, beyond mitigating the adverse effects of OFO, SIF may also regulate lipid metabolism by activating key signaling pathways. Research has indicated that SIF can act as a ligand for PPARα and activate the AKT/mTORC1 signaling pathway, thereby promoting fatty acid β-oxidation and inhibiting lipogenesis, which ultimately reduces lipid accumulation [37]. However, the specific functional targets and pathway mechanisms by which SIF regulates lipid metabolism in the *M. albus* remain to be further elucidated.

Serum levels of GOT and GPT are critical biomarkers of liver damage. The results of this experiment indicated that chronic dietary exposure to OFO significantly elevated the serum concentrations of GOT and GPT, suggesting hepatic injury, which was further corroborated by histopathological analysis (H&E staining). In contrast, supplementation with soy isoflavones was found to effectively mitigate the hepatic injury induced by the OFO.

Free radicals like ROS are molecules possessing unpaired electrons [38]. They arise from internal metabolic processes, such as mitochondrial electron leakage [39], and from external sources, notably the consumption of oxidatively spoiled foods [40]. Oxidative stress ensues when ROS generation exceeds cellular antioxidant capacity, directly damaging vital macromolecules. This damage manifests as lipid peroxidation, protein carbonylation, and DNA lesions [41,42,43], which in turn impairs cellular function, contributes to disease. This research demonstrated that dietary administration of OFO induces hepatic oxidative stress in the *M. albus*. This finding is consistent with the research by Changyou Song et al. in Wuchang bream (*Megalobrama amblycephala*), which reported that the ingestion of OFO decreased hepatic GSH content and led to a substantial accumulation of ROS and MDA [44]. The mechanisms by which OFO induces hepatic oxidative stress may involve multiple pathways. Firstly, the process of FO oxidation itself generates a large quantity of exogenous ROS. Upon entering the liver, these exogenous ROS can interfere with mitochondrial function, particularly by increasing electron leakage at complexes I and III of the respiratory chain, thereby further promoting the generation of endogenous ROS [45]. Secondly, the aldehyde end-products abundant in OFO can significantly deplete non-enzymatic antioxidants within the organism, such as GSH [46]. However, following the supplementation with SIF, the expression of *nrf2* in the liver of the *M. albus* was significantly upregulated, and the antioxidant capacity was enhanced. This aligns with the findings of Bo Yang in *Ctenopharyngodon idella*, where a diet containing 50 mg/kg of SIF was found to significantly upregulate *nrf2* expression and promote the expression of *sod* and *cat* [47]. This suggests that SIF may bolster the antioxidant defenses of the organism via the engagement of the *nrf2* signaling pathway.

The maintenance of organismal antioxidant homeostasis is intricately linked to the preservation of ER function. The findings of this study indicated that the intake of OFO induces ERS in the liver of the *M. albus*. This effect is likely associated with the excessive accumulation of ROS mediated by oxidative stress. ROS can inhibit the function of the ER calcium pump [48], impeding the reuptake of Ca^2+^ into the ER lumen and thereby disrupting ER calcium homeostasis. The depletion of luminal calcium further impairs the efficiency of protein folding, consequently triggering ERS [49]. Notably, the experimental results also revealed that the supplementation of SIF attenuated ERS, a phenomenon that may be closely attributable to its potent antioxidant properties.

The synergistic effect of oxidative stress and ERS can trigger an inflammatory cascade. This study found that the intake of OFO induced a hepatic inflammatory response. This observation is in agreement with the findings of Junliang Luo et al. [50], which showed that OFO induces systemic inflammation in a white shrimp (*Litopenaeus vannamei*) model. The underlying mechanism likely involves the combined activation of hepatic oxidative stress and ERS. On one hand, ROS accumulation can directly trigger the *nfkb* transcriptional program, which promotes the secretion of pro-inflammatory cytokines [51]. Concurrently, the three UPR sensors—*perk*, *ire1*, and *atf6*—may synergistically activate the *nfkb* signaling cascade from the ERS pathway [52]. Notably, dietary supplementation with SIF effectively alleviated the hepatic inflammatory state. This protective effect may originate from the ability of SIF to block the *nfkb*-mediated pro-inflammatory signaling network by enhancing the organism’s antioxidant defense capacity and inhibiting the aberrant activation of ERS.

## 5. Conclusions

Long-term dietary administration of OFO induces hepatic injury and growth inhibition in the *M. albus*. These adverse effects are characterized by an accumulation of serum TC and TG, diminished intestinal digestive enzyme activity, and elevated levels of hepatic oxidative stress and ERS, which ultimately culminate in an inflammatory response and liver damage. Conversely, supplementation with SIF was found to effectively mitigate these detrimental changes. The findings of this study indicate that a dietary inclusion of 50 mg/kg SIF is the most efficacious dosage based on overall performance.

## Figures and Tables

**Table 1:** Composition and nutrient level of experimental diet (%, dry matter).

Ingredients	CON	SIF0	SIF25	SIF50	SIF100
^1^ Fish meal	40.00	40.00	40.00	40.00	40.00
Soy protein concentrate	16.00	16.00	16.00	16.00	16.00
Poultry by-product meal	3.00	3.00	3.00	3.00	3.00
Beer yeast	5.00	5.00	5.00	5.00	5.00
^2^ Oxidized fish oil	0.00	2.20	2.20	2.20	2.20
^3^ Fish oil	2.20	0.00	0.00	0.00	0.00
Microcrystalline cellulose	10.26	10.26	10.26	10.26	10.26
Corn gluten meal	20.00	20.00	20.00	20.00	20.00
Choline	0.50	0.50	0.50	0.50	0.50
Ca(H_2_PO_4_)_2_	2.00	2.00	2.00	2.00	2.00
^4^ Premix	1.00	1.00	1.00	1.00	1.00
^5^ Antioxidants	0.01	0.01	0.01	0.01	0.01
^6^ Mold inhibitor	0.03	0.03	0.03	0.03	0.03
^7^ Soy isoflavones	0.00	0.00	25.00 mg/kg	50.00 mg/kg	100.00 mg/kg
^8^ Proximate composition (%)					
Crude protein	41.36	40.74	40.91	41.45	41.17
Crude lipid	5.96	5.78	6.02	5.84	6.09

Note: ^1^ Peruvian fishmeal, purchased from Zhangjiajie Xinrui Co., Ltd. ^2^ The value of POV is 831 meq O_2_/kg ^3^ Peruvian fish oil, purchased from Zhangjiajie Xinrui Co., Ltd. ^4^ Provided by MGO Ter Bio-Tech Co., Ltd. (Qingdao, China). Vitamin and Mineral Premix composition (mg/kg diet): KI (1%) 100 mg, CoCl_2_·6H_2_O (1%) 50 mg, CuSO_4_·5H_2_O 4 mg, FeSO_4_·H_2_O 120 mg, ZnSO_4_·H_2_O 60 mg, MnSO_4_·H_2_O 150 mg, Na_2_SeO_3_·5H_2_O (1%) 10 mg, MgSO_4_·H_2_O 30 mg, VB1 5 mg, riboflavin 8 mg, VB6 6 mg, VB12 0.02 mg, VK3 5 mg, inositol 100 mg, pantothenic acid 20 mg, niacin acid 30 mg, folic acid 1.7 mg, biotin 0.05 mg, VA 15 mg, VD3 0.375 mg, VE 40 mg, VC 100 mg; ^5^ The main component of antioxidant is ethoxyquin; ^6^ The main component of mould inhibitor is calcium propionate; ^7^ Purchased from Chengdu Yuancheng Technology Co. ^8^ The crude protein and crude lipid were measured.

**Table 2:** Real-time PCR primer sequences.

Gene	Accession No.	Forward Primer (5′-3′)	Reverse Primer (5′-3′)
*rpl 17*	XM_020587712.1	CGAGAACCCGACTAAATCA	GTTGTAGCGACGGAAAGG
*gpx1*	XM_020607739.1	AGATGTGAATGGGAAGGATGCC	AAACTTCGGGTCAGTCATCAGG
*nrf2*	XM_020596408.1	TCACAGACGAGAATGATGCC	CTGCTACTGGGAACTGAAACTG
*gpx8*	XM_020593975.1	ATCCTGCCTTCAGATTCCTCAC	TCATTTCTCGCACCAGCACT
*sod*	XM_020598412.1	GTTGCCAAGATAGACATCACGG	TCATTGCCTCCTTTTCCCAG
*cat*	XM_020624985.1	CATTGGGAAGACTACACCTATCGC	GATGAAGAAGATGGGGGTGTTG
*grp78*	XM_020600810.1	ACCTGACTGGCATCCCTCCTG	CTTGTTCTTGTTGCCTGTGCC
*atf6*	XM_020607104.1	AACCGTCGTGGTGACACTTTC	GTCCGTCACTTCACAGTCAATC
*xbp1*	XM_020623950.1	ATGGTGGTAGTAACAGCAGGGAC	CTGCGACTCTGTTCTTTAGTTTCC
*atf4*	XM_020586222.1	TCTTTCACGGGCATGGATTG	GAGAATCGTCGGATGAGCAAG
*eif2α*	XM_020603841.1	AGCCCCTTCCTTTGTTCGTC	CTGCTGAGGCTTTCTTGTTCCAC
*ire1*	XM_020619837.1	TTCGAGAACGCAACCGTATCA	CATCATCCATACCGCTATCATTC
*perk*	XM_020604189.1	CTCCCTACAGTCAAATGGAAGCC	AGGGGAAGTAGTAACCGTTGTC
*pparγ*	XM_020609689.1	CAAAGCCTCTGGGTTTCACTAT	TTTGTTGCGGGACTTCTTGT
*tgf-β1*	XM_020605575.1	AAGTCCAGCAAGCAATCCCTAG	GAGATGCTTGTTGGGTCCTTG
*tgf-β3*	XM_020590885.1	GCCAAGAAAAACGAACAGAGG	TGTCACATCAAAGGAGACCCAC
*nfkb*	KC841853.1	CTTCGTAACCCAGAGGATAAACC	CAGATAAACACTGCACAGCCAAG
*il-1β*	KM262825.1	GTCAACCTCATTATCGCCACG	AAACTCCTCTTCTGGCTGTCG
*il-8*	XM_020596483.1	CAACTCCCACTGCAAAGATACTG	CGACTTTGCCAGTTTCCTTTC
*tlr-8*	XM_020596483.1	TTTGTCCTAACAGAGGGCTACG	CAGCATCAGCAGCACAATCAC

**Table 3:** Growth data for each experimental group of *M. albus* (*n* = 3).

Item	CON	SIF0	SIF25	SIF50	SIF100	*p*_Values
^1^ IBW	26.01 ± 0.02	25.97 ± 0.03	26.01 ± 0.01	25.98 ± 0.02	25.97 ± 0.01	anova: *p* = 0.47
^2^ FBW	54.15 ± 2.21 ^a^	47.8 ± 0.46 ^b^	52.87 ± 0.15 ^a^	56.43 ± 0.8 ^a^	54.38 ± 0.18 ^a^	anova: *p* < 0.05
^3^ WGR	108.05 ± 8.62 ^a^	84.13 ± 1.62 ^b^	102.36 ± 1.13 ^ab^	111.8 ± 6.0 ^a^	106.76 ± 0.86 ^a^	anova: *p* < 0.05
^4^ SR	93.33 ± 1.76	93.0 ± 1.73	96.0 ± 0.0	97.0 ± 0.58	97.0 ± 0.58	anova: *p* = 0.08
^5^ SGR	1.3 ± 0.07 ^a^	1.09 ± 0.02 ^b^	1.26 ± 0.01 ^ab^	1.34 ± 0.05 ^a^	1.3 ± 0.01 ^a^	anova: *p* < 0.05
^6^ CF	0.11 ± 0.0 ^ab^	0.1 ± 0.0 ^b^	0.12 ± 0.0 ^a^	0.13 ± 0.0 ^a^	0.12 ± 0.0 ^a^	kruskal: *p* < 0.05

Note: In the same row, different superscripted lowercase letters indicate significant differences (*p* < 0.05). ^1^ IBW (g): Initial body weight; ^2^ FBW (g): Final body weight; ^3^ WGR (%): Weight gain rate = 100 × (FBW − IBW)/IBW; ^4^ SR (%): Survival rate = 100 × final number/initial number; ^5^ SGR (%/d): Specific growth rate = 100 × [ln(FBW) − ln(IBW)]/days; ^6^ CF (100 g/cm^3^): Condition factor = 100 × body weight/(body length)^3^.

## Data Availability

The data of this study are available from the corresponding author upon reasonable request.
